# Preparation for Rural Practice with a Multimodal Rural Emergency Medicine Curriculum

**DOI:** 10.5811/westjem.18573

**Published:** 2024-11-18

**Authors:** Ashley K. Weisman, Skyler A. Lentz, Julie T. Vieth, Joseph M. Kennedy, Richard B. Bounds

**Affiliations:** UVM Health Network, The Robert Larner M.D. College of Medicine at the University of Vermont, Department of Emergency Medicine, Burlington, Vermont

Rural regions face emergency physician shortages. Most training programs are located in cities and lack rural clinical experiences, didactics, and mentorship to excite and prepare residents for rural emergency medicine (EM) practice. There is limited data on optimal training methods for preparing residents for rural practice. To address this need for rural EM training and workforce, we developed a rural EM curriculum for a single EM residency program in the United States. We began with a two-year case review from critical access emergency departments. Rural EM skills were defined and taught using lectures, simulation cases, and clinical rotations. We obtained quantitative and qualitative feedback from the first 10 residents participating in the curriculum. Qualitatively, 10/10 residents gained new skills and found these experiences valuable to their training and career choice, with 100% expressing interest in rural practice and 70% choosing a rural practice. Quantitatively, residents managed a wide variety of patient acuity and volume and performed new procedures compared to their academic center rotations, all while gaining unique skills from the challenges of a rural environment. Focused rural EM clinical experiences and nonclinical teaching during residency are a promising approach to bridge the gap between urban, tertiary-care training programs and rural emergency care needs.

## BACKGROUND

Rural regions face significant emergency physician (EP) shortages.^1^ ^,^ ^2^ As rural patients age and face even greater disparities and barriers to care,^3^ ^–^ ^6^ the need for skilled, rural EPs prepared to practice full spectrum emergency medicine (EM) with limited resources is paramount. Recent EM workforce studies demonstrate that the critical need for rural EPs is likely to worsen rather than improve for several reasons.^7^ ^–^ ^10^ There is a significant mismatch in the distribution of EPs throughout the United States, with a surplus of EPs in urban areas and a deficit in rural areas.^9^ Rural areas have lower rates of physician entry, an older workforce, and higher rates of attrition.^10^ Most important for the future of the rural EM workforce, EM training programs continue to proliferate in urban areas, with very few programs in rural areas with rural-focused training.^7^ ^,^ ^8^

Several training programs have sought to encourage rural practice by incorporating rural clinical electives and published guidelines for developing rural clinical experiences.^11^ ^,^ ^12^ Additional research demonstrates that rural rotations are educationally valuable and include greater or similar numbers of procedures to urban tertiary rotations.^13^ Despite these efforts, a significant educational gap remains for most residents. There is no requirement for rural EM clinical or nonclinical education within the Accreditation Council for Graduate Medical Education core competencies for EM and no standardized rural EM curriculum for residencies to reference. There is also very limited data on optimal training methods for preparing residents for rural practice.

## OBJECTIVES

Our team of rural EM clinician-educators is well versed in the staffing needs and clinical demands of rural EM practice. We aimed to leverage our unique, rural-academic health network and address this educational challenge. Our objectives were to 1) deliver a multimodal (didactic, simulation, and clinical) rural EM curriculum that prepares trainees to independently work in rural emergency departments (ED) at graduation; and 2) evaluate our program quantitatively and qualitatively to assess the opportunities and limitations of rural training.

## CURRICULAR DESIGN

To develop our curriculum content, rural EM needed to be defined: What are the rural-specific skills that EPs need to deliver excellent care that are not part of a typical tertiary-care residency curriculum? To answer this question, we conducted a two-year case review of all admissions, transfers, and greater than six-hour length-of-stay (LOS) cases from the EDs of two rural critical access hospitals (CAH) in our network. To be designated “critical access,” a hospital must be located in a rural area more than 35 miles from another hospital, provide 24/7 emergency care, and have 25 or fewer inpatient beds.^14^ The two CAH EDs in our study each see 6,000 patients annually with basic EM resources including lab, plain radiographs, computed tomography, and point-of-care ultrasound, with a solo coverage EP staffing model. There is no specialty back-up, no operating room, no obstetrical services, no blood bank, no respiratory therapy, and limited pharmacist coverage.

We focused on cases requiring critical care, transfer, specialty consultation, and greater than six-hour LOS, because these cases pushed our attending group to think harder, review a procedure or pathology, or use skills typical of not only an EP, but also a respiratory therapist, pharmacist, and/or specialist. This allowed us to identify a library of challenging rural EM cases. We also created a 15-member rural EM faculty working group of academic physicians who routinely practice in rural environments. Our rural CAH case review was combined with input from our rural EM working group to create a list of knowledge and skills essential to rural EM practice yet missing from our existing teaching (Figure). In parallel, we developed a selection of EM clinical rotations at our network’s CAHs and at partner rural sites throughout the country.

**Figure.**
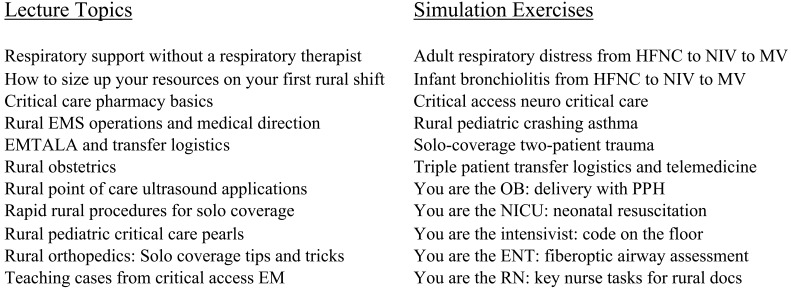
Nonclinical rural curriculum components.

*EMS*, emergency medical services; *EMTALA*, Emergency Medicine Treatment and Labor Act; *HFNC*, high-flow nasal cannula; *NIV*, non-invasive ventilation; *MV*, mechanical ventilation; *IO*, intraosseous; *OB*, obstetrician; *PPH*, post-partum hemorrhage; *NICU*, neonatal intensive care unit; *ENT*, ear nose and throat; *RN*, registered nurse.

**Table.** Rural emergency medicine curriculum participation and resident job choice.

Resident group	Number of residents	Rural curriculum completed	Rural practice
Home resident, pilot	10	Clinical and nonclinical	70% (7/10)
Home resident, other elective	8	Nonclinical	75% (6/8)
Visiting resident	4	Clinical	25% (1/4)

Ten residents completed a pilot two-week or four-week rural EM rotation and 18 months of the rural nonclinical curriculum from July 2021–August 2023. These 10 home residents were the first 10 residents to choose this rural rotation in their second or third year as part of a new residency program. During the study period, eight other home residents chose alternative elective rotations. All 18 home residents completed the rural nonclinical curriculum. During the study period, we also opened our rural EM rotation to visiting residents. Four visiting residents from three urban residency programs completed the clinical rotation but did not participate in the nonclinical curriculum. Zero residents entered our rural curriculum with an explicit commitment to future rural practice. One home resident in each group and two visiting residents grew up in a rural area—defined as a United States census-designated place with a population less than 2,500.^15^

Qualitative and quantitative feedback on the rural rotation was solicited from home and visiting residents. Residents completed a voluntary survey that asked the following: 1) Was the rotation beneficial to your clinical training; 2) describe any new skills and unique experiences; 3) discuss any limitations of learning in a rural environment; 4) are you interested in rural practice; and 5) where will you practice upon graduating? We also collected data on patient volume, patient acuity (discharged, admitted, or transferred), and procedures performed from April 2022–August 2023. Prior to April 2022, our electronic health record did not consistently enable resident documentation, preventing accurate chart review. This quality improvement and education project using research methods was submitted to our institutional review board (IRB); it did not meet the definition for human subjects research and was exempt from IRB review and approval.

## IMPACT/EFFECTIVENESS

The rural EM curriculum has proven successful over the first three years and continues to evolve. A total of 10 of 10 home residents and four of four visiting residents who completed the clinical rotation reported gaining new skills and confidence. In their free-text responses, residents stated that providing respiratory support without a respiratory therapist, managing multiple critical patients, providing solo-coverage trauma care, and delivering orthopedic care were highlighted skills of the rotation. Compared with academic, tertiary-care sites, residents see patient volumes that are more variable at rural sites with more extreme peaks (six patients per hour) and troughs (zero patients per hour) consistent with solo coverage EM practice. Acuity is similarly more variable than at academic sites on a given shift, but high-acuity cases were noted to be some of the highest yield learning experiences with residents performing full-spectrum emergency care. Residents performed a greater variety of procedures than at their tertiary site and more new procedures. These were procedures they often deferred to specialists on busy shifts including fracture-dislocation reductions, arthrocentesis, complex laceration repair, priapism reduction, and regional anesthesia. A total of 100% of home and visiting residents reported interest in future rural practice, and 70% of home residents who completed the clinical and nonclinical rural curriculum signed a contract with a rural CAH or sole community hospital^16^ for a portion of their practice on graduation (Table). Additionally, 75% of home residents who completed the nonclinical curriculum only also chose a rural practice.

As of 2024, this curriculum continues to evolve based on resident and faculty feedback. Data collection on the impact of rural EM education in our program is ongoing.

## LIMTATIONS

This single-center pilot study of a new curriculum within a rural health network comes with inherent limitations. For measuring impact, no comparison group was available. As a new residency program, there are no prior classes of residents to compare outcomes pre- and post- rural curriculum. All home residents completed the rural nonclinical curriculum and received mentorship from rural faculty. Additionally, five of the eight residents who did not choose the pre-designed rural EM rotations created their own rural/austere rotation. Thus, our study does not isolate clinical or nonclinical components to determine which might be more strongly associated with our residents’ high rate of rural practice following graduation.

For generalizability, many programs may lack easy access to rural clinical sites, mentorship from rural faculty, or rural lecture/simulation resources. With innovation and collaboration, however, rural clinical and nonclinical education should be feasible for any program. All programs have didactic time, access to simulation training, and patients received in transfer from rural sites in their region. Clinical experiences could be created through partnerships with other programs if not available locally. To support other programs’ rural-focused education, we are developing a free, open-access “Rural EM Resource” website (ruralemresource.us) to share our lectures, simulation cases, and template for building rural clinical rotations.

## CONCLUSION

A multimodal, rural curriculum represents a feasible approach to preparing residents for independent practice in rural settings. Innovative rural curricula should be shared broadly, and urban residency programs should partner with rural clinical sites to provide expanded training opportunities. This type of focused rural clinical and nonclinical curriculum development represents a promising approach to bridging the gap between urban, tertiary-care training programs and rural emergency care needs.
